# Effect of Orange-Fleshed Sweet Potato Purée and Wheat Flour Blends on β-Carotene, Selected Physicochemical and Microbiological Properties of Bread

**DOI:** 10.3390/foods11071051

**Published:** 2022-04-06

**Authors:** Derick Malavi, Daniel Mbogo, Mukani Moyo, Lucy Mwaura, Jan Low, Tawanda Muzhingi

**Affiliations:** 1 Food and Nutritional Evaluation Laboratory (FANEL), International Potato Center (CIP), Nairobi 25171-00603, Kenya; d.m.mbogo@greenwich.ac.uk (D.M.); mukani.moyo@cgiar.org (M.M.); l.mwaura@cgiar.org (L.M.); j.low@cgiar.org (J.L.); tmuzhin@ncsu.edu (T.M.); 2 Department of Food Technology, Safety and Health, Faculty of Bioscience Engineering, Ghent University, Coupure Links 653, 9000 Ghent, Belgium; 3 Food Chemistry and Technology Research Centre, Department of Food Technology, Ghent University Global Campus, 119-5, Songdomunhwa-ro, Yeonsu-gu, Incheon 21985, Korea; 4 Natural Resources Institute (NRI), Medway Campus, University of Greenwich, Central Avenue, Chatham ME4 4TB, UK; 5 Department of Food, Bioprocessing and Nutrition Sciences, North Carolina State University, Raleigh, NC 27695, USA

**Keywords:** orange-fleshed sweet potato, purée, composite bread, β-carotene, vitamin A deficiency, biofortification

## Abstract

Partial substitution of wheat flour with orange-fleshed sweet potato (OFSP) purée in bread can increase vitamin A intake among consumers. The study investigated the influence of wheat flour substitution with 20–50% of OFSP purée on proximate composition, color, β-carotene, water activity, and microbial keeping quality. The moisture content, crude protein, crude fat, total ash, crude fiber, and carbohydrate in bread ranged from 28.6–32.7%, 9.9–10.6%, 5.0–5.5%, 1.9–3.2%, 1.4–1.8%, and 79.1–80.9%, respectively. β-carotene, total ash, and crude fiber contents in bread, and Hunter color values a*, b*, chroma, and ∆*E* significantly increased with the addition of OFSP purée. Total viable counts (TVC), yeast, and molds in bread ranged from 2.82–3.64 log_10_ cfu/g and 1.48–2.16 log_10_ cfu/g, respectively, on the last day of storage. Water activity, TVC, and fungal counts were low in sweet potato composite bread as compared to white bread. Total β-carotene in OFSP bread ranged from 1.9–5.4 mg/100 g (on dry weight). One hundred grams of bread portion enriched with 40% and 50% OFSP purée provides more than 50% of vitamin A dietary requirements to children aged 4–8 years. Incorporation of up to 50% OFSP purée in wheat flour produces a relatively shelf-stable, nutritious, and health-promoting functional bread.

## 1. Introduction

Bread is a global staple food that plays a vital role in food security [1]. Over the past decades, the change in food habits has tremendously increased the demand for wheat-based foods, such as bread, in developing countries [2]. Wheat flour is the main raw material for the processing of bread. It has proteins with superior baking properties [3]. Regardless of the unique qualities of wheat flour proteins for bread-making, wheat flour is low in health-promoting bioactive compounds, such as beta-carotene [4]. Wheat flour contains high amounts of digestible starch [5] with a high glycemic index, making wheat bread unsuitable for consumption by patients suffering from diabetes, hypertension, and other cardiovascular diseases [6]. Furthermore, the soaring prices of wheat on the global market and concerns over the increase in importation in sub-Saharan Africa warrant the interest to partially substitute it in bakery applications. This would reduce dependency on wheat imports and lower the cost of production for bakeries and consequently generate more profits.

Vitamin A is an essential micronutrient for human health. It is primarily involved in vision, reproduction, growth, and cellular communication [7,8]. Vitamin A deficiency (VAD) is a prevalent health problem in sub-Saharan Africa (SSA) and South Asia. It results from consumption of diets low in bioavailable vitamin A [9,10]. Limited access to diets rich in vitamin A has been a major factor contributing to the high prevalence of VAD among populations in SSA [11,12]. VAD is one of the leading causes of childhood blindness, morbidity, and mortality from infections in children and pregnant women in low and middle-income countries [12]. According to the World Health Organization Database, approximately 190 million preschool children and pregnant women suffer from VAD worldwide [12]. A previous study by [9] has further reported 40% of children below five years are deficient in vitamin A.

Strategies, such as dietary diversification, vitamin A supplementation, and food fortification, are being explored to address vitamin A deficiency. These approaches are however not feasible and sustainable [12]. Further, animal food sources rich in vitamin A, such as dairy products, fish, and meat, are not consumed regularly by the majority of poor households in developing countries [10,11]. Alternatively, these households obtain about 82% of their total dietary vitamin A from plant-based foods [12]. For these reasons, plant-based foods and biofortified crops, such as sweet potatoes, offer cheap and sustainable alternatives for alleviating VAD [10,13]. Among the carotenoids, β-carotene is the most important provitamin A carotenoid. It is found in fruits, leafy green vegetables, orange, and yellow vegetables [8,10].

Biofortified orange-fleshed sweet potato (OFSP) is being grown and promoted as a food-based strategy to address the problem of VAD in SSA [11,14]. OFSP is rich in beta-carotene, a precursor for Vitamin A. An ex-ante assessment by [13] demonstrated that OFSP could largely contribute to fighting VAD in SSA. A different study by [10] recommends OFSP as a long-term strategy in combating VAD in children in SSA. About 100–125 g of boiled or steamed OFSP adequately provides the recommended dietary intake (RDA) of retinol equivalent per day in children below five years [10,13,15,16]. Unlike many vegetables, OFSP is also a good source of energy, antioxidants, vitamins C, E, K, B vitamins, and minerals such as phosphorus, potassium, and dietary fiber [17].

OFSP is bulky, highly perishable, and mainly consumed as boiled or steamed in SSA. However, the majority of the urban population has a low preference towards boiled sweet potato [18]. Efforts to expand the processing and utilization of OFSP in SSA are still in progress. It is currently processed into a purée for partial wheat flour substitution in the production of bread by bakeries in SSA [19,20]. The purée is prepared by washing, peeling, steaming, and mashing sweet potatoes which retain more than 90% of beta-carotene. Conventionally, the production of flour by drying has been a practical approach for enhancing the shelf-life of sweet potatoes. Although this saves on problems related to perishability, storage and transportation, significant loss in carotenoids occurs [21,22]. Regardless, several studies have reported the use of OFSP flour as an ingredient in baked goods [3,21,23,24,25,26]. Therefore, the incorporation of OFSP purée in foods is a more ideal approach for potentially improving vitamin A dietary intake [27] in Kenya and SSA in general. A study by [28] revealed a high preference and willingness to pay for OFSP bread among consumers in urban areas. Thus, OFSP bread can help in fighting VAD in children, pregnant women, and lactating mothers in urban areas [29].

Bakeries in SSA are currently substituting wheat flour with up to 30% of OFSP purée in the production of sweet potato bread [19]. However, the potential to increase the current substitution level to produce a more nutritious composite bread exists. A few studies have reported sweet potato purée as an ingredient in baked goods in regard to their sensory attributes, physical properties, consumer preference, and contribution to vitamin A [27,28,29,30,31]. A recent study by [5] found that increasing the proportion of OFSP purée concentration (10 to 50%) in bread improves the bioacccessibility of β-carotene and alters the digestion of starch, beneficial for human health. However, there is limited information on the effect of substituting wheat flour with different proportions of OFSP purée on nutritional and some physicochemical properties of sweet potato bread.

The shelf-life of bread is relatively short. It is limited mainly by staling and spoilage by microorganisms, such as *Bacillus subtilis*, yeast, and molds. There has also been an attempt to assess the shelf-life of bread substituted with 30% OFSP purée [31] but more information on different levels of substitution is required. OFSP flour has recently been shown to decrease staling in wheat–OFSP composite bread [3]. On the contrary, OFSP purée can support microbial growth and act as a source of contamination in bread if good hygiene practices are not observed [32], hence the need for microbial quality assessment. To date, there is no information on the influence of OFSP purée proportion on microbial quality and shelf-life of bread.

The objectives of this study, therefore, were: (1) to develop sweet potato composite bread from different proportions of wheat flour and OFSP purée, and (2) to investigate the effects of wheat flour substitution with 20–50% of OFSP purée on proximate composition, β-carotene, color, water activity and microbial shelf-life of the composite bread.

## 2. Materials and Methods

### 2.1. Materials

Sweet potato purée was prepared from fresh OFSP roots of the “VITA” variety at the purée processing company in Homa Bay County, Kenya. It was prepared following methods described by [33,34]. The sweet potato roots were washed, hand-peeled, trimmed, and cut into 0.5 cm-thick slices using a mechanical slicer. Then, the slices were steamed for half an hour at 100 °C. The cooked sweet potato was cooled for 1 h on trays and comminuted into a purée using a puréeing machine (OMAS Food Machinery, AEE1T0, Euro ingredients limited, Oggiona con Santo Stefano, Italy). The purée was vacuum packed in 5 kg plastic bags and immediately stored in the freezer (−20 °C). The frozen purée was delivered to International Potato Centre (CIP-Nairobi) in ice-cold boxes and stored at −20 °C before analysis.

All the ingredients: commercial baker’s flour (United Millers Limited, Kisumu, Kenya, protein content 10% on dry matter basis); bread improver (VITABAKE D 300, Pack Ingredients, Nairobi, Kenya), formulated with corn starch, ascorbic acid, calcium carbonate, and baking enzymes (α-amylase and hemicellulase); salt (Kensalt Ltd., Mombasa, Kenya), sugar (West Sugar Co. Limited, Kakamega, Kenya), baker’s yeast (Dalet Foods, Nairobi, Kenya), and cooking fat (Bidco, Africa Ltd., Thika, Kenya) were purchased from a retail outlet in Nairobi. All the ingredients were purchased from a retail chain store in Nairobi and formulated as shown in Table 1. Wheat flour was mixed with OFSP purée in the ratios of 100:0, 80:20, 70:30, 60:40, and 50:50. These were used to prepare 20, 30, 40, and 50% sweet potato purée bread. White bread was prepared with 100% wheat flour as a control for the study.

### 2.2. Bread Making

The bread was produced by straight dough according to [31,35] with modifications. The dough for each treatment was prepared in three replicates while maintaining the processing conditions for both sweet potato bread and wheat flour bread (control). Yeast was dissolved in warm water at 30 °C, followed by mixing of the dry ingredients (Table 1). Melted fat, sweet potato purée, and other ingredients were then mixed (Tombak bakery and confectionary F2 mixer). The ingredients were mixed for 3 min at low speed, followed by 5 min mixing at high speed. After complete mixing, the dough was weighed, cut into 450 g portions, and molded into rolls. The molded doughs were placed in baking tins smeared with a layer of oil and proofed at 30 °C for 45 min at 85% relative humidity. The proofed dough was baked in a preheated oven (FİMAK Rotary Oven, Turkey) at 230 °C for 30 min [24]. The loaves of bread were depanned and cooled on clean and sanitized shelves at (23 ± 1 °C) for 2 h [36]. The bread samples were vacuum-packed and aseptically transferred to the laboratory for analysis.

### 2.3. Sample Preparation

The bread was sliced manually using sterilized kitchen knives. Nine slices were selected from each bread for analysis. They were equally selected from the center, and both ends of each bread. The crust was manually cut and separated from the crumb and grinded separately for beta-carotene analysis. The crust (20 mm × 20 mm × ~4 mm) and the crumb (20 mm × 20 mm × 20 mm) were also prepared manually for color measurement. Samples for proximate composition and water activity analyses were milled without the separation of the crumb and the crust. Bread samples for the analysis of water activity and microbial profile were analyzed immediately while the rest of the samples were kept in a clean and sanitized oven space at 25 °C to mimic the normal shelf storage conditions for bread.

Sample preparation, extraction, and analysis were performed under golden-yellow lights to prevent the degradation of beta-carotene. All the samples were analyzed for moisture content, protein, fat, fiber, ash, β-carotene, color, water activity, total viable counts, and fungal counts. All the analyses were performed in triplicate.

### 2.4. Determination of Proximate Composition in Bread

Moisture content, crude fat, crude protein, crude fiber, and total ash were determined according to the Official Methods of Analysis of AOAC International [37].

#### 2.4.1. Determination of Moisture Content

The moisture content was determined by the method of AOAC 934.01 [37]. Two grams (2 g) of well-homogenized bread samples were transferred to the dried weighed dishes. The samples on the dishes were dried in the oven for 2 h at 105 °C until achieving a constant weight. The dried samples were removed from the oven, cooled in a desiccator at room temperature, and reweighed:

(1) $$Moisture\;\left(\%\right)=\frac{\left(W_{1}-W_{2}\right)\times 100}{S_{w}}$$

where *W*_1_ is the weight of the dish and fresh sample, *W*_2_ is the weight of the dish and dried sample, and *S_w_* is the sample weight.

#### 2.4.2. Determination of Total Ash Content

The ash content was determined according to the methods of AOAC 942.05 [37]. Two grams (2 g) of the sample were weighed and placed into a dry crucible of predetermined weight. The sample-containing crucibles were charred on a hot plate and placed in a muffle furnace at 550 °C. The samples were ignited for 6 h. The samples were removed from the furnace, cooled in a desiccator at room temperature, and weighed:

(2) $$Total\;Ash\;\left(\%\right)=\frac{\left(W_{1}-W_{2}\right)\times 100}{S_{w}},$$

where *W*_1_ is the weight of the ash and crucible after incineration, *W*_2_ is the weight of the empty crucible, and *S_w_* is the initial weight of the sample.

#### 2.4.3. Determination of Crude Fat

The fat content was performed by acid hydrolysis and Solvent extraction method as described by AOAC 920.39 [37]. Five grams (5 g) of the sample were weighed in a 300 mL hydrolysis tube and hydrolyzed with 100 mL of 4 N HCl at 170 °C for 1 h. The samples were filtered, dried in an oven (at 70 °C) for 8 h. The dried samples were transferred into extraction thimbles and extracted for fat using SER 148 Solvent Extractor (VELP Scientific, Italy) with petroleum ether as the extraction solvent. The receiver flask was then dried in the oven at 100 °C for 30 min to evaporate traces of the solvent. The flask was cooled in the desiccator to room temperature and reweighed:

(3) $$Crude\;Fat\;\left(\%\right)=\frac{\left(W_{f}-W\right)\times 100}{S_{w}},$$

where *W_f_* is the weight of the receiver flask and extracted fat, *W* is the weight of the empty receiver flask, and *S_w_* is the weight of the sample used.

#### 2.4.4. Determination of Crude Fiber

The crude fiber content in bread was established according to the AOAC 978.10 methods [37]. Two grams of defatted sample were transferred into a 1 L beaker and digested on a hot plate with 25 mL of 2.5 M H_2_SO_4_ for 30 min. The volume was maintained at 200 mL with hot distilled water while stirring the sample during the digestion process. It was then filtered through the glass wool on a Buchner funnel. The second digestion step was conducted using 2.5 M NaOH. The sample was filtered again through the same glass wool. The residue on the glass wool was rinsed with 3 mL of ethanol. The glass wool together with the residue were dried in an oven at 105 °C for 30 min until a constant weight (*W*_1_) was achieved. The oven-dried samples were then incinerated in a muffle furnace at 600 °C for 3 h. The incinerated sample was cooled in a desiccator at room temperature and reweighed:

(4) $$Crude\;Fiber\;\left(\%\right)=\frac{\left(W_{1}-W_{2}\right)\times 100}{S_{w}},$$

where *W*_1_ is the weight of the crucible and sample before incineration, *W*_2_ is the weight of the crucible containing ash, and *S_w_* is the weight of the sample.

#### 2.4.5. Determination of Crude Protein

Protein content was performed by the Kjeldahl method as described by AOAC [37]. Exactly 0.5 g of the bread sample was weighed into 100 mL digestion tubes, and 10 mL of concentrated H_2_SO_4_ was added with 2 g of Kjeldahl tablet (catalyst). Samples were digested until a pure colorless solution was obtained. Kjeldahl distiller was used for distillation with 35% NaOH (*w/w*) to release ammonia from the sample. The distilled ammonia was collected in a flask with 50 mL of 4% boric acid mixed with bromocresol green and methyl red indicators. The distilled sample was titrated against 0.1 HCl. The protein content was calculated by multiplying the value of nitrogen by a factor of 5.7 [38]:

(5) $$Crude\;protein\;\left(\%\right)=\frac{\left(a\times b\times 14\times 5.7\right)\times 100}{W}$$

where *a* is the normality of the acid, *b* is the volume of the standard acid used (mL), *W* is the weight of the sample (g), and 5.7 is the conversion factor for protein from % nitrogen.

#### 2.4.6. Available Carbohydrates

The percentage of available carbohydrates was calculated by subtracting the total contents of moisture, total ash, crude protein, crude fat, and crude fiber content from 100%:

(6) Available carbohydrate=100−(Moisture (%)+Protein (%)+Fat (%)+Ash (%)+Fiber (%))

#### 2.4.7. Gross Food Energy

The gross energy content of bread was estimated by Atwater’s conversion equation [39]:

(7) $$Food\;energy\left(\frac{kcal}{g}\right)=\left(\%AC\times 4\right)+\left(\%TF\times 9\right)+\left(\%CP\times 4\right)\;$$

where *AC* is the available carbohydrate, *TF* is total fat, and *CP* is the crude protein.

#### 2.4.8. Quality Control

All the proximate composition analyses were performed in triplicate. Cereal-based food (FC 290, PT-FC-770) from LGC Standards Proficiency Testing UK, previously used for proficiency testing in the lab, and an in-house quality control food material (wheat bran), were used as reference materials for quality control.

### 2.5. Extraction and Quantification of Beta-Carotene

#### 2.5.1. Extraction of β-Carotene in OFSP Purée Composite Bread

β-carotene extraction in bread was conducted by saponification and extraction with hexane as described by [40] with modifications. Approximately 2 g of each sample (crust and crumb) was weighed separately in 25 mL screw-capped glass tubes. Then, 6 mL of ethanol (with 0.1% butylated hydroxytoluene (BHT)) and 250 µL of echinenone (internal standard) were added. The samples were further homogenized, vortexed for 1 min, and incubated in a water bath (SW23GB, JULABO) at 85 °C for 10 min. This was followed by the addition of 120 µL of 80% KOH (*w/v*) to each tube, mixing, and further incubation at 85 °C for 5 min. The tubes with the samples were removed from the water bath and immediately cooled in a bucket full of ice. Distilled water (5 mL) was added to each tube while still immersed in ice and vortexed for 1 min. This was followed by the addition of 3 mL hexane (≥99.8% HPLC grade, Sigma-Aldrich, St. Louis, MO, USA,) and mixing on the vortex mixer for 1 min. The tubes were then centrifuged (Eppendorf, Centrifuge 5810) at 3000 rpm for 10 min. The upper phase of hexane was extracted using a Pasteur pipette into separate clean 25 mL Pyrex glass test tubes. The extraction and separation processes were repeated thrice by adding 3 mL hexane to each sample pellet in the tubes. β-carotene extraction was confirmed to be complete after obtaining a white residue and a clear upper liquid phase. The extract was washed by addition of 5 mL of distilled water, vortexing, and centrifugation at 3000 rpm for 5 min. The upper hexane phase was separated into 15 mL clean test tubes and evaporated under a stream of nitrogen using a nitrogen evaporating machine (N-Evap machine, Organomation, Model OA-8125, River Rd, Berlin, MA, USA) with a water bath at 40 °C. Dried tubes with carotenoids were then reconstituted with 5 mL of methanol and tetrahydrofuran (HPLC grade) (85:15 *v/v*) and filtered through a 0.2 µm PFTE syringe filter into amber vials for HPLC analysis.

#### 2.5.2. Extraction of Beta-Carotene in Orange-Fleshed Sweet Potato Purée

β-carotene extraction in OFSP purée was performed according to [41] with some modifications. Approximately 1 g of the sample was weighed (in triplicate) in a 25 mL test tube, followed by the addition of 5 mL methanol and 250 µL of the internal standard. The sample was vortexed for 1 min and incubated at 70 °C for 10 min. The sample was vortexed, centrifuged for 10 min (at 3000 rpm), and the upper phase was transferred into 25 mL volumetric flask. Tetrahydrofuran (THF) (5 mL) was added to the sample in the tube, mixed on the vortex for one minute, centrifuged, and separated. The extraction was repeated three times. The flask was filled to the mark with THF and mixed on the vortex for 1 min. Two milliliters (2 mL) of the extract were transferred to a separate tube and washed by adding 3 mL of distilled water, 4 mL of hexane, and 0.5 mL of methanol. The samples were centrifuged for 3 min at 3000 rpm. The upper phase (hexane) was separated and dried in a stream of nitrogen using the N-Evap machine. The dried tubes were reconstituted with 2 mL of methanol and THF (HPLC grade) (85:15 *v/v*) and filtered through a 0.2 µm PFTE syringe filter into 2 mL-amber vials for HPLC analysis.

#### 2.5.3. HPLC Conditions

Ten microliters (10 µL) of each sample were injected into the HPLC (Waters Alliance, 2695 Separations Module, Milford, MA, USA) with an auto-sampler and a UV-Vis photodiode array detector (PDA-2996). Empower software controlled the entire system and data acquisition. The extracts were resolved on a 150 mm × 4.6 mm, YMC C30, 3 µm particle size HPLC column (Wilmington, NC, USA) utilizing a reverse-phase gradient HPLC method. The mobile phases were methanol/tert-butyl methyl ether/water (85:12:3, *v/v/v*, with 1.5% ammonium acetate in the water-mobile phase A) and methanol/tert-butyl methyl ether/water (8:90:2, *v/v/v*, with 1% ammonium acetate in the water-mobile phase B). A linear gradient elution was employed and set as follows: 0–1 min, 100% A; 1–10 min, 90% A; 10–22 min, 45% A; 22–33 min, 5% A; 33–37 min, 5% A; 37–39 min, 100% A; 39–40 min, 100% A. The flow rate was maintained at 0.4 mL per minute and a run time of 40 min [40].

#### 2.5.4. Quantification of β-Carotene in Purée and Bread

All-*trans*, 13-*cis*, and 9-*cis* β-carotene in the samples were identified based on their absorption spectra and retention time of their respective pure standards. Quantification was performed using the calibration curves of pure β-carotene standards after correcting for extraction efficiency based on recovery levels of echinenone [40].

#### 2.5.5. Contribution of OFSP Purée–Wheat Composite Bread to Vitamin A Requirement

Retinol activity equivalents (RAE) were calculated according to the conversion factor of 12 µg of β-carotene to 1 µg of retinol [42]. Vitamin A activity of the *cis*-isomers of β-carotene was estimated to be half of the *trans*-β-carotene [21,43]. The contribution towards the recommended daily vitamin A body requirement to different groups: children (4–8 years), adolescents (10–18 years), adult males and females (19–65 years), and pregnant and lactating mothers was based on a portion of 100 g for each bread sample.

### 2.6. Determination of Color in Bread

Color measurement was based on Hunter L* (a measure of lightness), a* (a measure of greenness to redness), and b* (a measure of blueness to yellowness) values. The color of bread was determined using a Hunter color meter (Lovibond LC100, Parkland Dr, Sarasota, FL, USA) with standard illuminant D_65_, observation angle of 10°, and an aperture of 30 mm [21,36,44]. The Hunter color values for the crust (20 mm × 20 mm × ~4 mm) and the crumb (20 mm × 20 mm × 20 mm) of bread were determined separately by pressing the chroma meter against the transparent glass petri dish (60 × 15 mm) filled with approximately 100 g of the sample. The instrument was calibrated using a standard white reference tile with values L* = 98.63, a* = 0, and b* = 0. Three readings were recorded from different positions of the petri dish with the sample. All the samples were analyzed in three replicates. The psychometric color terms chroma, hue angle, and delta *E* were calculated according to the methods of [45,46], as follows:

(8) $$Chroma=\sqrt{a^{2}+b^{2}}$$

(9) $$Hue\;angle\;\left(h^{{}^{\circ}}\right)=tan^{-1}\left(\frac{b}{a}\right),$$

(10) $$ƊE=\sqrt{{\left(L_{1}-L_{0}\right)}^{2}+{\left(a_{1}-a_{0}\right)}^{2}+{\left(b_{1}-b_{0}\right)}^{2}},$$

where *L*_0_, *a*_0_, and *b*_0_ are Hunter color values of white bread and *L*_1_, *a*_1_, and *b*_1_, are experimental color values of different sweet potato composite breads.

### 2.7. Determination of Water Activity in Bread

Water activity (aw) was determined by a water activity meter (Aqua Lab Series 4TE, Decagon Devices, Inc., NE Hopkins Ct., Pullman, WA, USA) [31]. The instrument was calibrated with 0.5 mol/kg, aw = 0.984 potassium chloride standard solution at 25 °C. Water activity was determined after 0, 3, and 6 days of storage. The analysis was performed in triplicate.

### 2.8. Microbial Analysis of Bread

Total mesophilic (total viable bacterial counts) and fungi were carried out to determine the microbial load of the bread samples by pour plate method according to [47,48] with modifications. Briefly, twenty-five grams (25 g) of each bread sample was mixed with 225 mL of sterile buffered peptone water, agitated vigorously, and allowed to settle. A ten-fold subsequent serial dilution series was performed with 0.85% saline water (NaCl) to obtain a colony count of 1 × 10^−3^/mL. Precisely 0.1 mL of each serial dilution was spread evenly and separately on Petri dishes with solidified plate count agar (PCA) for total counts and potato dextrose agar (PDA) for enumeration of yeasts and molds. All the analyses were performed in triplicate. Cultured PCA plates were incubated at 30 °C for 48 h, while PDA plates were incubated at 25 °C for five days. The colonies were enumerated and recorded after incubation. Microbial quality evaluation was conducted on day 0, day 3, and day 6 of storage. The analyses were performed in triplicate.

### 2.9. Statistical Analysis

The experimental data were subjected to statistical evaluation using analysis of variance (ANOVA) followed by Tukey’s test to determine the significant differences between bread quality characteristics of the various formulations. Pearson correlation was also performed to establish the relationship between the variables. The analyses were conducted using the statistical software for social sciences (SPSS 26.0, SPSS, Inc., Chicago, IL, USA). The statistical significance was defined as (*p* < 0.05). Principal Component Analysis (PCA) was performed using The Unscrambler X (Version 10.4, Oslo, Norway) to distinguish the bread samples based on their attributes.

## 3. Results and Discussion

### 3.1. Proximate Composition of Baked Bread

Table 2 shows the proximate composition of the sweet potato purée, wheat flour, and sweet potato composite bread.

Moisture content is an essential food quality attribute that largely contributes to the physical, sensory, and microbial properties of bread. The moisture content of bread in this study varied significantly (*p* < 0.05), with the lowest value in white bread (28.6 g/100 g) and the highest in 50% OFSP purée bread (32.7 g/100 g). The range of moisture content in the current study is similar to the findings by [31,49]. Other studies by [36,50] have reported a higher moisture content of 36.6 to 41.6% and 32.8–37.4%, respectively, in sweet potato flour composite bread, contrary to findings in this study. The high moisture content in sweet potato bread is attributed to the high binding water capacity of sweet potato starch associated with weak molecular forces between the starch granules [51]. This avails more molecular surfaces for binding water during starch gelatinization and ultimately higher retained moisture content in baked bread [52,53]. On the other hand, the relatively low moisture content in white bread can be due to the low binding capacity of starch in wheat flour resulting in excessive loss of moisture during baking [53].

Lipids from wheat flour, shortenings, and dough strengtheners, influence dough development, fermentation, proofing, baking, and bread storage [54]. On the other hand, dietary fats and oils contribute to the taste of sweet potato bread and enhance the bio-accessibility of β-carotene [55,56,57]. The crude fat content of bread in this study ranged from 5.0 to 5.5%, and it was not significantly different (*p* > 0.05). Our findings report a similar range of crude fat content as [50]), but higher as compared to [26], which reported a range of 2.3–3.7% in sweet potato–wheat flour composite bread. The fat content in the purée was relatively low (0.6%). Therefore, an increase in OFSP purée to wheat flour substitution ratio had an insignificant effect (*p* > 0.05) on the fat content of bread.

The protein content in the bread ranged from 9.9 g/100 g in 20% OFSP bread to 10.6 g/100 g in white bread. Similar findings in sweet potato flour composite bread have been reported by [26]. On the contrary, the protein content in our study was higher than the results by [50,58]. The protein in sweet potato composite bread slightly increased with an increase in substitution levels, but it was not significant (*p* > 0.05).

The crude fiber content was significantly (*p* < 0.05) higher in OFSP bread as compared to the white bread. The fiber content of sweet potato composite bread in the current study ranged from 1.2 to 1.8%. This range was higher in comparison to bread formulated by wheat, maize, and sweet potato flours in a study by [24]. The fiber content in our study is however lower compared to a range of 1.8–4.0% reported in wheat flour bread supplemented with green vegetable powders [58]. Similarly, a higher amount of fiber has been reported in wheat–sweet potato flour composite bread [26]. The differences in fiber content are due to varietal and fiber compositional differences among different wheat flour substitutes. Regardless of the slightly lower fiber content in OFSP bread in comparison to other studies, this study reveals OFSP bread as an additional food source for fiber. Consumption of OFSP bread is therefore beneficial as it can provide dietary fiber essential for human health. Dietary fiber increases satiety and promotes gastrointestinal peristalsis, hence alleviating constipation, facilitating weight loss, and lowering cholesterol levels in the blood [59,60].

The ash content is equivalent to the total mineral content in foods. OFSP purée was high in ash, and an increase in supplementation ratio increased the total ash content in bread. The ash content in the present study varied from 1.9% in white bread to 3.2% in 50% sweet potato purée bread. This range is comparable to reported ash contents of 1.4–3.0% and 2.3–3.3% in sweet potato flour composite bread reported by [24,26], respectively. It is however necessary to investigate further specific minerals, such as iron and zinc in sweet potato composite bread.

Available carbohydrate and gross food energy contents in bread ranged from 79.1–80.9% and 407.0–414.5 kcal/100 g, respectively. The carbohydrate content in bread in this study was high compared to other studies [50,58]. A high level of carbohydrate in the form of starch is desirable for gelatinization and bread texture development. The inclusion of sweet potato purée bread in the diet can substantially contribute to the daily energy requirements necessary for metabolic functions.

### 3.2. Beta-Carotene Content and Retinol Activity Equivalents (RAEs) in OFSP Purée Composite Bread

Table 3 shows β-carotene content and RAEs in sweet potato purée and sweet potato purée bread. β-carotene was undetected in wheat flour bread. Total β-carotene in wheat–OFSP composite bread ranged from 1.9–5.4 mg/100 g. Our results are similar to previously reported study findings by [21,27]. The concentration of β-carotene significantly (*p* < 0.05) increased with an increase in the proportion of OFSP purée.

**Figure 1:**
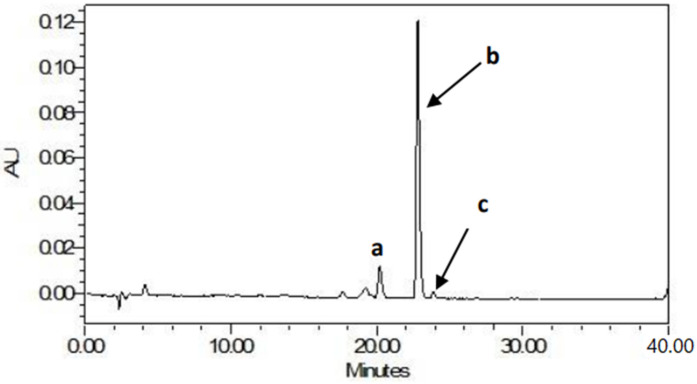
HPLC chromatogram showing beta-carotene peaks in the extract of orange-fleshed sweet potato composite bread at 450 nm. Peak identification: (a) 13-*cis* β-carotene, (b) all-trans ß-carotene (c) 9-*cis* β-carotene.

A typical HPLC chromatogram of the carotenoids in bread from OFSP variety “VITA” obtained with the YMC C30, 3 µm particle size HPLC column is presented in Figure 1, demonstrating the predominance of β-carotene. The *trans*- and *cis*-isomers of β-carotene were detected in OFSP purée bread. More than 70% of the total β-carotene in bread was in *trans* form, which has more vitamin A activity compared to the *cis*-isomers. The *trans*- and *cis*-β-carotene in bread significantly (*p* < 0.05) increased with an increase in the percentage of OFSP purée. The concentration of the *13-cis*, all-*trans* and *9-cis* β-carotene in sweet potato composite bread ranged from 1410–3950 µg/100 g, 430–1200 µg/100 g, and 70–240 µg/100 g, respectively.

As a rule of thumb, processed foods containing at least 15 µg/g can be considered a good source of vitamin A [27]. In the current study, bread formulated with 30%, 40%, and 50% OFSP purée contained more than 15 µg/g of *trans*-β-carotene, hence a good source of vitamin A. As expected, OFSP bread contained more of the *cis*-β-carotene and less of the *trans*-β-carotene in comparison to fresh OFSP purée. This is in agreement with [15,21]. The baking process induces isomerization of the *trans*-β-carotene, thereby increasing the concentration of the *cis*-form in bread.

### 3.3. Contribution of Vitamin A Daily Requirement to Different Age Groups by OFSP Purée Bread

Retinol Activity Equivalents (RAEs) (Table 3) provided by sweet potato bread from different treatments and their percentage contribution towards vitamin A daily body requirement in different groups is indicated in Table 4.

OFSP bread in this study could provide 138.5–389.1 µg/100 g of RAEs. RAE significantly (*p* < 0.05) increased with an increase in the ratio OFSP purée and wheat flour. Consumption of 100 g of 30% sweet potato bread could provide about 47% and 32% of vitamin A daily body requirement to adolescents and children below five years, respectively. A similar amount could provide about 25% and 15% of vitamin A RDA to pregnant and lactating women, respectively. OFSP bread (50%) can provide up to 97% of vitamin A daily body requirement to children below the age of five, 65% to adolescents, 51% to pregnant women, and 30% to lactating mothers.

### 3.4. Color of OFSP Purée Composite Bread

Bread color is an important attribute that influences consumer acceptability. Wheat flour and sweet potato purée blend ratio influenced the color of bread (Table 5). The lightness (L*), redness (*a*)*, yellowness (b*), chroma, hue angle, and ∆*E* values of the crumb significantly (*p* < 0.05) varied from 65.3–77.8, 1.0–11.4, 17.8–40.7, 17.9–42.2, 74.3–86.6, and 11.0–28.1, respectively. L*, a*, b*, chroma, *h*°, and delta E values for the crust varied from 57.3–69.2, 7.7–13.4, 25.4–35.7, 26.6–38.1, 66.3–73.1, and 9.8–16.7, respectively and were significantly different (*p* < 0.05). L* and hue angle values for both the crust and crumb significantly decreased with the addition of the purée in bread, while a*, b*, chroma, and ∆*E* values increased significantly (*p* < 0.05). A recent study by [3] has also reported similar findings in bread composited with OFSP flour. The partial substitution of wheat flour with sweet potato purée produced redder, yellower, and brighter bread as indicated by the ∆*E* values. The high ∆*E* values of sweet potato bread were high, indicating the color changes could easily be perceived without a closer visual inspection in comparison to wheat flour bread. The color change is contributed by β-carotene from the OFSP purée, Maillard browning, and caramelization, which are influenced by the distribution of water and the reaction between reducing sugars and amino acids [35].

### 3.5. Water Activity of Sweet Potato Purée Bread

**Figure 2:**
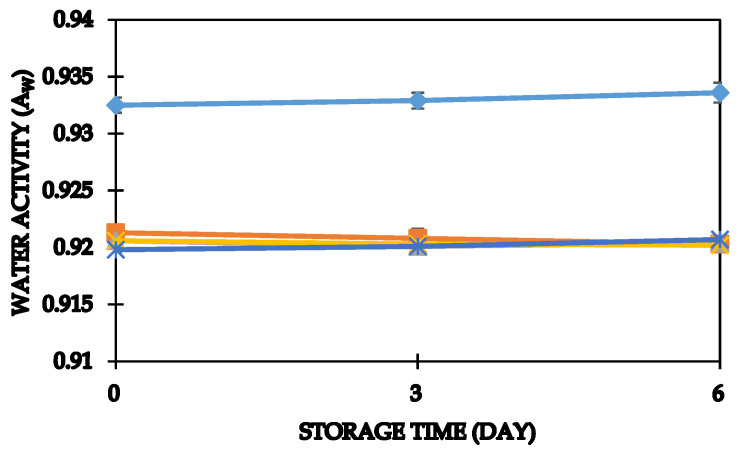
Water activity profile in bread during storage. The bars indicate standard errors of means. White bread (♦); 20% OFSP bread (■); 30% OFSP bread (▲); 40% OFSP bread (∗); 50% OFSP bread (∗).

Water activity is a significant factor that influences microbial growth in foods [61]. Besides influencing crumb firmness, water activity is a vital storage factor for bread [62]. Water activity (aw) in OFSP purée bread was significantly (*p* < 0.05) lower as compared to white bread. Water activity in bread from the current study ranged from 0.92–0.93 (Figure 2).

Our study findings were in agreement with water activity range values in bread from previous studies by [31,36]. Water activity slightly decreased in sweet potato bread, but a gradual increase was observed in white bread over the storage period. The authors of [31,62] have reported a reduction in aw in bread formulated with hydrocolloids and OFSP purée, respectively. Though insignificant (*p* > 0.05), the decline in water activity in sweet potato bread can be attributed to the concentration of natural sugars in the purée due to loss of moisture during storage.

### 3.6. Microbial Keeping Quality of OFSP Purée Bread

**Figure 3:**
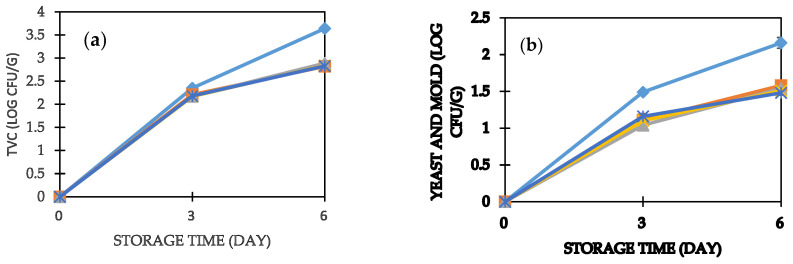
Changes in: (**a**) total viable counts (TVC); (**b**) yeast and molds in bread during storage. The bars indicate standard errors of means. White bread (♦); 20% OFSP bread (■); 30% OFSP bread (▲); 40% OFSP bread (∗); 50% OFSP bread (∗).

The microbial quality profiles of bread in the study are shown in Figure 3a,b. Microbial counts in sweet potato purée bread were significantly lower (*p* < 0.05) in comparison to the control sample. TVC, yeast, and molds were absent in freshly baked bread. This shows that the bread was of good quality and safe for consumption. Total counts ranged between 2.17–2.35 log_10_ cfu/g and 2.82–3.64 log_10_ cfu/g after 3 and 6 days of storage, respectively.

Yeast and molds cause surface growth, discoloration, and off-flavors in bread [36]. From the findings, there is a tendency of OFSP purée limiting the growth of yeast and molds in wheat–OFSP composite bread. The fungal counts ranged from 1.10–1.49 log_10_ cfu/g and 1.48–2.16 log_10_ cfu/g after three and six days of storage, respectively. These counts are low compared to a study by [31] but high compared with findings from other studies [36,63]. Mold growth characterized by black, yellow, and green coloring clusters were observed on white and OFSP bread after 3 and 6 days of storage, respectively. These could potentially have been *Aspergillus*, *Penicillium, Rhizopus,* and *Mucor* species. There was a significant (*p* < 0.01) positive correlation between water activity and microbial counts in bread. The high microbial counts and fast growth of molds on white bread are attributed to the high water activity. Besides the low water activity, OFSP purée bread has a relatively long shelf life due to the increased moisture retention, hence reducing the rate of staling.

### 3.7. Correlation of Physical and Nutritional Attributes of Bread

The correlation matrix in Table 6 shows the relationship of different nutritional and the selected physical parameters of bread. A significant (*p* < 0.01) positive correlation existed between moisture content and ash (r = 0.885), beta-carotene (r = 0.910) and Hunter color values a* (r = 0.886), b* (0.876), chroma (0.881), and ∆E (0.904). On the contrary, a significant (*p* < 0.01) moderate negative correlation existed between moisture content and carbohydrate (r = −0.75) and water activity (r = −0.630). Sweet potato composite bread higher in moisture content/low dry matter will tend to be high in ash and β-carotene. It is a challenge to simultaneously improve dry matter and beta-carotene among the sweet potato germplasm during biofortification. The positive correlation between β-carotene and total ash suggests an indirect improvement of the latter through selection for higher β-carotene in the variety used for the study [64].

There was a significant (*p* < 0.01) negative correlation between L* (a measure of lightness) and a*, b*, c*, ∆*E*, and β-carotene content in bread (r = −0.908, −0.806, −0.898 and −0.926, respectively). The findings illustrate that bread with a high proportion of wheat flour would be expected to be lighter in color due to low amounts of β-carotene. The amount of β-carotene in bread, positively correlated (*p* < 0.05) with Hunter color values a* (r = 0.979), b* (r = 0.940), c* (r = 0.946) and ∆*E* (r = 0.924). Similar findings have been reported in a previous study by [21]. The color parameters a* and b* can be useful in predicting the amount of β-carotene in processed products with OFSP as a natural colorant.

### 3.8. Principal Component Analysis (PCA)

**Figure 4:**
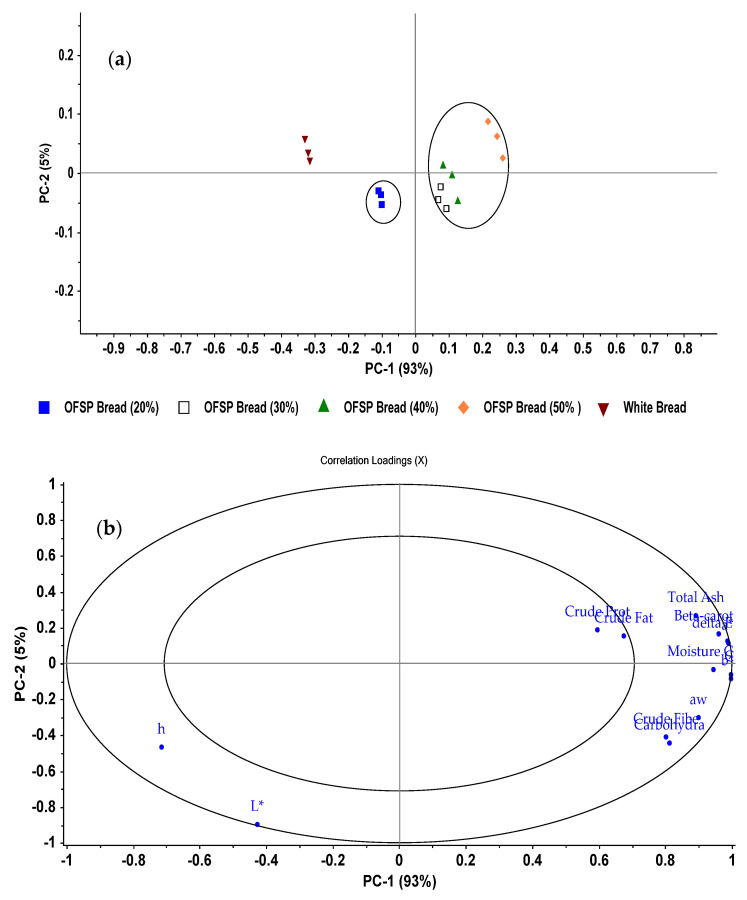
(**a**) Score plots, and (**b**) correlation loading plots from the principal component analysis of wheat−OFSP purée bread quality attributes.

The correlation loading and score plots from the Principal Component Analysis (PCA) of wheat–OFSP composite bread are shown in Figure 4a,b, respectively. From the findings, the first two principal components (PC1 and PC2) explained 98% of the variance in the original data. A large percentage of the variance was explained by PC1 (93.0%), whereas the second PC accounted for 5% of the variance. From Figure 4a, PCA analysis clearly discriminated breads based on their physical and chemical attributes. White bread and 20% sweet potato bread were clustered on side of the PC 1. Breads with purée at 30%, 40%, and 50% were clustered on the positive quadrants of PC1.

As indicated by the correlation plot (Figure 4b), the variation in PC1 was largely characterized by Hunter color values a*, b*, chroma, and ∆*E*, β-carotene, and moisture content of bread. These components were highly correlated to each other as indicated by their location on the outer ellipse of the correlation loadings. Water activity (aw), total ash, crude fiber also contributed to the variation in PC1. These attributes were prominent in sweet potato purée breads. On the other hand, crude protein and crude fiber were located in the inner circle of the correlation loadings. They, therefore, do not contain enough structured variation to distinguish different bread formulations in the study. The variation in PC2 was contributed by color parameters L* and hue angle (*h*°). They positively correlated to each other (r = 0.848) and contributed to the PC2 variation that separated white bread from sweet potato purée composite bread.

## 4. Conclusions

The current study reports the effect of partially substituting wheat flour with OFSP purée on the nutritional, selected physical and microbial quality of sweet potato purée composite bread. An increase in the proportion of OFSP purée as a substitute for wheat flour significantly increased the β-carotene, total ash, and fiber contents of bread. Sweet potato purée also enhanced the color and microbial keeping quality of bread. Consumption of a 100 g portion of 40% and 50% OFSP bread can provide 76% and 97% of vitamin A daily requirement in children below five years. A similar portion of bread formulated with 50% OFSP purée provides half (51%) and 30% of vitamin A daily requirement in pregnant women and lactating mothers, respectively. However, further studies should be considered to determine the dough characteristics, texture properties, and sensory attributes of sweet potato bread.

## Figures and Tables

**Table 1:** Ingredient formulation for wheat flour–OFSP purée composite bread (g/kg).

	Wheat Flour	OFSP Purée	Fat	Sugar	Salt	Flour Improver	Yeast	Water
White bread (0:100)	1000	0	25	30	12	2	10	666
OFSP bread (20:80)	800	200	25	20	12	2	10	361
OFSP bread (30:70)	700	300	25	20	12	2	10	315
OFSP bread (40:60)	600	400	25	20	12	2	10	200
OFSP bread (50:50)	500	500	25	20	12	2	10	100

g denotes weight in grams.

**Table 2:** Proximate composition of raw ingredients and OFSP purée–wheat flour composite bread (DWB) ^1^.

Treatment	Moisture (%)	Crude Fat (%)	Crude Protein (%)	Crude Fiber (%)	Ash (%)	Available Carbohydrate (%)	Energy (kcal/100 g)
OFSP purée	67.5 ± 0.4 ^f^	0.6 ± 0.1 ^a^	5.2 ± 0.0 ^a^	5.0 ± 0.2 ^c^	4.3 ± 0.1 ^e^	84.9 ± 1.2 ^c^	365.5 ± 4.7 ^a^
Wheat flour	10.4 ± 0.3 ^a^	2.3 ± 0.1 ^b^	10.2 ± 0.1 ^b^	1.4 ± 0.1 ^a^	1.8 ± 0.0 ^a^	73.9 ± 1.0 ^a^	357.1 ± 3.2 ^a^
White bread (0:100)	28.6 ± 0.9 ^b^	5.4 ± 0.0 ^c^	10.6 ± 0.0 ^b^	1.2 ± 0.1 ^a^	1.9 ± 0.1 ^a^	80.9 ± 1.2 ^b^	414.5 ± 4.7 ^b^
OFSP bread (20:80)	29.1 ± 0.5 ^b,c^	5.0 ± 0.0 ^c^	9.9 ± 0.2 ^b^	1.8 ± 0.0 ^b^	2.4 ± 0.0 ^b^	80.5 ± 0.8 ^b^	406.6 ± 2.5 ^b^
OFSP bread (30:70)	30.8 ± 0.7 ^c,d^	5.1 ± 0.1 ^c^	10.0 ± 0.2 ^b^	1.8 ± 0.1 ^b^	2.5 ± 0.0 ^b^	80.6 ± 1.1 ^b^	407.6 ± 5.1 ^b^
OFSP bread (40:60)	31.9 ± 0.5 ^d,e^	5.4 ± 0.3 ^c^	10.4 ± 0.2 ^b^	1.7 ± 0.1 ^b^	2.8 ± 0.2 ^c^	79.6 ± 1.1 ^b^	408.7 ± 1.9 ^b^
OFSP bread (50:50)	32.7 ± 0.8 ^e^	5.5 ± 0.2 ^c^	10.4 ± 0.5 ^b^	1.8 ± 0.2 ^b^	3.2 ± 0.1 ^d^	79.1 ± 1.8 ^b^	407.0 ± 5.1 ^b^

^1^ Each value is expressed as mean ± SD (*n* = 3). Mean values with different superscript letters within the same column are significantly different (*p* < 0.05). DWB: Nutritional composition except for moisture, is expressed on a dry weight basis.

**Table 3:** Beta-carotene content (mg/100 g) and Retinol Activity Equivalents (RAE) (µg/100 g) in OFSP purée–wheat flour composite bread (DWB) ^1^.

OFSP Purée: Wheat Flour (%)	β-Carotene Crust	β-Carotene Crumb	13-*cis* β-carotene	All-*trans* β-Carotene	9-*cis* β-Carotene	Total β-Carotene	RAE ^b^
White bread (0:100)	nd	nd	nd	nd	nd	nd	-
OFSP bread (20:80)	0.8 ± 0.1 ^a^	1.2 ± 0.0 ^a^	0.43 ± 0.00 ^a^	1.41 ± 0.06 ^a^	0.07 ± 0.00 ^a^	1.9 ± 0.1 ^a^	138.5 ± 7.0 ^a^
OFSP bread (30:70)	1.0 ± 0.0 ^b^	1.6 ± 0.1 ^b^	0.61 ± 0.00 ^b^	1.92 ± 0.07 ^b^	0.11 ± 0.01 ^b^	2.6 ± 0.1 ^b^	189.4 ± 4 ± 5.6 ^b^
OFSP bread (40:60)	1.5 ± 0.1 ^c^	2.7 ± 0.0 ^c^	0.94 ± 0.01 ^c^	3.07 ± 0.03 ^c^	0.19 ± 0.01 ^c^	4.2 ± 0.0 ^c^	302.8 ± 2.8 ^c^
OFSP bread (50:50)	1.7 ± 0.1 ^d^	3.7 ± 0.1 ^d^	1.20 ± 0.26 ^d^	3.95 ± 0.05 ^d^	0.24 ± 0.01 ^d^	5.4 ± 0.1 ^d^	389.1 ± 4.7 ^d^

^1^ Each value is expressed as mean ± SD (*n* = 3) and means with different superscript letters within the same column are significantly different (*p* < 0.05). DWB-nutritional composition is expressed on a dry weight basis, nd—not detected, RAE (Retinol Activity Equivalents) calculated by the conversion factor: 12 µg all-trans β-carotene = 1 µg retinol and 24 µg of cis-β-carotene = 1 µg retinol. Total RAE from each bread sample was obtained by the summation of conversion values by all-trans and cis beta-carotene [42,43].

**Table 4:** Percentage contribution of vitamin A RDA in different groups by OFSP purée–wheat flour composite bread ^a^.

OFSP Purée: Wheat Flour (%)	Children 4–8 Years (400 ^b^)	Adolescents 10–18 Years (600 ^b^)	Adult Males 19–65 Years (900 ^b^)	Adult Females 19–65 Years (700 ^b^)	Pregnant Women (770 ^b^)	Lactating Women (1300 ^b^)
White Bread (0:100)	0.0	0.0	0.0	0.0	0.0	0.0
OFSP Bread (20:80)	34.6	23.1	15.4	19.8	18.0	10.7
OFSP Bread (30:70)	47.4	31.6	21.0	27.1	24.6	14.6
OFSP Bread (40:60)	75.7	50.5	33.6	43.3	39.3	23.3
OFSP Bread (50:50)	97.3	64.9	43.2	55.6	50.5	29.9

RDA: recommended daily allowances, ^a^ Values based on 100 g portion of bread on a dry weight basis, ^b^ RDA values adapted from [21,43].

**Table 5:** Color of OFSP purée and processed OFSP purée–wheat flour composite bread ^1^.

Sample	L*	a*	b*	c* (Chroma)	Hue (*h*°)	∆*E*
OFSP purée	54.3 ± 0.2 ^a^	19.1 ± 0.1 ^g^	44.1 ± 0.9 ^g^	48.1 ± 0.8 ^g^	66.6 ± 0.4 ^a^	-
White bread—crust	69.2 ± 0.2 ^f^	7.7 ± 0.2 ^c,d^	25.4 ± 0.8 ^b^	26.6 ± 0.8 ^b^	73.1 ± 0.1 ^c^	-
20% OFSP bread—crust	62.2 ± 0.2 ^d^	11.7 ± 0.2 ^e^	31.0 ± 0.8 ^c^	33.1 ± 0.8 ^d^	69.4 ± 0.5 ^b^	9.8 ± 0.6 ^a^
30% bread—crust	62.0 ± 0.1 ^d^	11.6 ± 0.2 ^e^	31.8 ± 0.5 ^d^	33.9 ± 0.6 ^d^	68.9 ± 0.2 ^b^	10.4 ± 0.4 ^a,b^
40% OFSP bread—crust	59.3 ± 0.2 ^c^	13.4 ± 0.2 ^f^	31.7 ± 0.2 ^d^	34.2 ± 0.1 ^d^	66.9 ± 0.5 ^a^	12.9 ± 0.2 ^c^
50% OFSP bread—crust	57.3 ± 0.1 ^b^	13.4 ± 0.8 ^f^	35.7 ± 0.5 ^e^	38.1 ± 0.2 ^e^	66.3 ± 0.3 ^a^	16.7 ± 0.5 ^d^
White bread—crumb	77.8 ± 1.1 ^i^	1.0 ± 0.1 ^a^	17.8 ± 0.4 ^a^	17.9 ± 0.6 ^a^	86.6 ± 0.3 ^h^	-
20% OFSP bread—crumb	75.8 ± 0.1 ^h^	5.0 ± 0.3 ^b^	27.9 ± 0.1 ^d^	28.3 ± 0.1 ^c^	79.8 ± 0.5 ^g^	11.0 ± 0.2 ^b^
30% OFSP bread—crumb	74.8 ± 0.3 ^h^	7.3 ± 0.2 ^c^	36.0 ± 0.2 ^e^	36.7 ± 0.5 ^e^	78.6 ± 0.2 ^f^	19.5 ± 0.4 ^e^
40% OFSP bread—crumb	71.5 ± 1.3 ^g^	8.4 ± 0.3 ^d^	36.2 ± 0.3 ^e^	37.1 ± 0.4 ^e^	76.9 ± 0.5 ^e^	20.8 ± 0.4 ^f^
50% OFSP bread—crumb	65.3 ± 0.7 ^e^	11.4 ± 0.6 ^e^	40.7 ± 0.4 ^f^	42.2 ± 0.6 ^f^	74.3 ± 0.9 ^d^	28.1 ± 0.2 ^g^

^1^ Each value is expressed as mean ± SD (*n* = 3) and means with different superscript letters within the same column are significantly different (*p* < 0.05). White bread with *L*_0_ = 69.2, *a*_0_ = 7.73, *b*_0_ = 25.4 for the crust and *L*_0_ = 77.8, *a*_0_ = 1.03, *b*_0_ = 17.8 for the crumb were used as standard reference values. ∆*E* is calculated based on formulae 10.

**Table 6:** Pearson correlation coefficients among physical and chemical attributes of bread.

	Moisture	Ash	Fat	Protein	Fiber	Carbohydrate	β-Carotene	L*	a*	b*	c*	*h*°	∆*E*	aw
Moisture	1													
Ash	0.879 **													
Fat	0.419	0.525 *												
Protein	0.277	0.260	0.594 *											
Fiber	−0.454	−0.350	−0.208	0.201										
Carbohydrate	−0.750 **	−0.486	−0.622 *	−0.696 **	−0.091									
β-carotene	0.910 **	0.915 **	0.332	0.048	−0.608 *	−0.296								
L*	−0.845 **	−0.939 **	−0.384	−0.172	0.403	0.356	−0.926 **							
a*	0.886 **	0.879 **	0.195	−0.035	−0.542 *	−0.220	0.979 **	−0.908 **						
b*	0.876 **	0.794 **	0.132	−0.087	−0.595 *	−0.187	0.940 **	−0.806 **	0.975 **					
c*	0.881 **	0.806 **	0.144	−0.077	−0.588 *	−0.194	0.946 **	−0.819 **	0.980 **	1.000 **				
*h*°	−0.824 **	−0.812 **	−0.077	0.179	0.598 *	0.108	−0.958 **	0.848 **	−0.982 **	−0.966 **	−0.968 **			
∆*E*	0.904 **	0.882 **	0.646 **	0.731 **	−0.063	−0.597 *	0.924 **	−0.898 **	0.981 **	0.985 **	0.991 **	−0.931 **		
aw	−0.630 *	−0.546 *	0.181	0.444	0.672 **	−0.107	−0.790 **	0.578 *	−0.832 **	−0.881 **	−0.874 **	0.913 **	−0.732 **	1

** Correlation is significant at the 0.01 level, * Correlation is significant at the 0.05 level.

## Data Availability

Not applicable.
